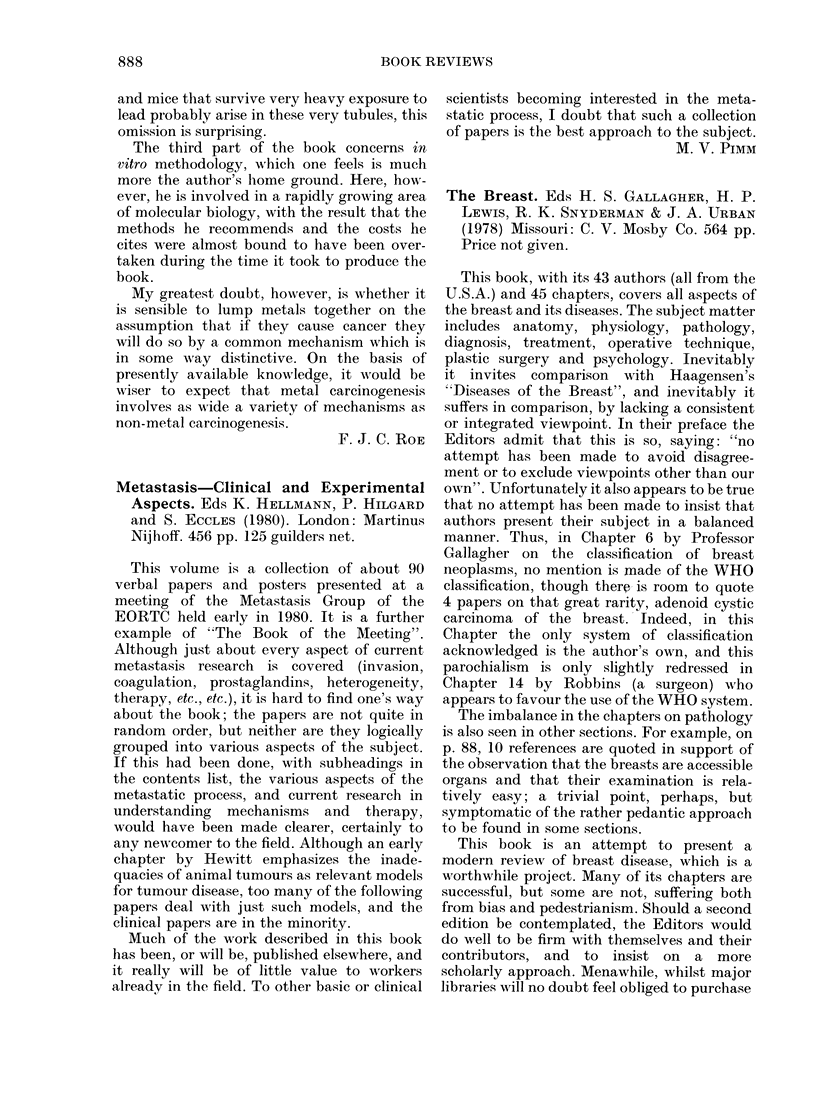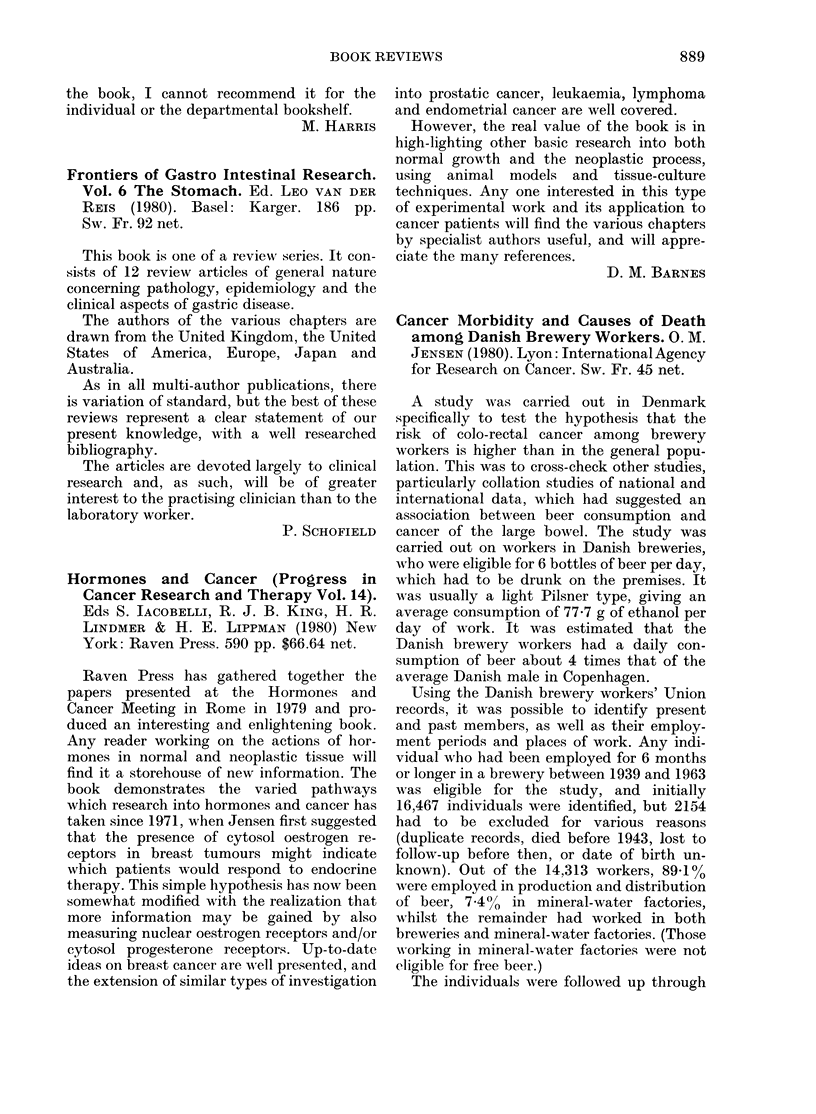# The Breast

**Published:** 1981-06

**Authors:** M. Harris


**The Breast.** Eds H. S. Gallagher, H. P. Lewis, R. K. Snyderman & J. A. Urban (1978) Missouri: C. V. Mosby Co. 564 pp. Price not given.

This book, with its 43 authors (all from the U.S.A.) and 45 chapters, covers all aspects of the breast and its diseases. The subject matter includes anatomy, physiology, pathology, diagnosis, treatment, operative technique, plastic surgery and psychology. Inevitably it invites comparison with Haagensen's "Diseases of the Breast", and inevitably it suffers in comparison, by lacking a consistent or integrated viewpoint. In their preface the Editors admit that this is so, saying: "no attempt has been made to avoid disagreement or to exclude viewpoints other than our own". Unfortunately it also appears to be true that no attempt has been made to insist that authors present their subject in a balanced manner. Thus, in Chapter 6 by Professor Gallagher on the classification of breast neoplasms, no mention is made of the WHO classification, though there is room to quote 4 papers on that great rarity, adenoid cystic carcinoma of the breast. Indeed, in this Chapter the only system of classification acknowledged is the author's own, and this parochialism is only slightly redressed in Chapter 14 by Robbins (a surgeon) who appears to favour the use of the WHO system.

The imbalance in the chapters on pathology is also seen in other sections. For example, on p. 88, 10 references are quoted in support of the observation that the breasts are accessible organs and that their examination is relatively easy; a trivial point, perhaps, but symptomatic of the rather pedantic approach to be found in some sections.

This book is an attempt to present a modern review of breast disease, which is a worthwhile project. Many of its chapters are successful, but some are not, suffering both from bias and pedestrianism. Should a second edition be contemplated, the Editors would do well to be firm with themselves and their contributors, and to insist on a more scholarly approach. Menawhile, whilst major libraries will no doubt feel obliged to purchase the book, I cannot recommend it for the individual or the departmental bookshelf.

M. Harris